# Long COVID syndrome in children: neutrophilic granulocyte dysfunction and its correlation with disease severity

**DOI:** 10.1038/s41390-024-03731-1

**Published:** 2024-11-27

**Authors:** Fanni Kovács, Tamás Posvai, Eszter Zsáry, Ferenc Kolonics, Réka Garai, Vivien Herczeg, Domonkos Czárán, Johanna Takács, Attila József Szabó, Péter Krivácsy, Roland Csépányi-Kömi

**Affiliations:** 1 Pediatric Center, MTA Center of Excellence, Semmelweis University, Bókay Unit, Bókay János Street 53-54, 1083 Budapest, Hungary; 2 Department of Physiology, Semmelweis University, Budapest, Hungary; 3 Centre for Translational Medicine, Semmelweis University, Budapest, Hungary; 4 Department of Social Sciences, Faculty of Health Sciences, Semmelweis University, Budapest, Hungary

## Abstract

**Background:**

Many children suffer from lingering symptoms after COVID-19, known as long COVID syndrome (LCS), otherwise called Post COVID-19 Condition (PCC). Despite extensive research, the prevalence of symptoms, its impact on quality of life, and underlying mechanisms still need to be fully understood. As neutrophilic granulocytes play an essential role in COVID-19, and their prolonged disruption was found to cause immunological diseases, we hypothesized their ongoing disturbed functionality in LCS.

**Methods:**

We studied 129 children with LCS, 32 convalescent children (CG+), and 8 uninfected children (CG−). Online questionnaires and in-person examinations assessed symptoms, quality of life, and functioning (QoL-F). Effector functions of neutrophilic granulocytes obtained from the venous blood of 29 LCS and 17 CG+ children were also investigated.

**Results:**

Persistent fatigue was the most common symptom in children with LCS, while both control groups complained about anxiety most frequently. LCS children experienced significantly more symptoms, impairing their QoL-F compared to CG+. Neutrophilic granulocyte dysfunction was found in LCS children, with decreased superoxide-producing activity and phagocytosis compared to CG+. The number of complaints of children with LCS correlated significantly with altered neutrophil effector functions.

**Conclusion:**

Neutrophil dysfunction in children with LCS may be part of the disease pathogenesis or a predisposing factor.

**Impact:**

- Using online questionnaires validated during in-person medical examinations and including two different control groups, our study compellingly supports and adds to previous clinical observations in the field.
- Our study provides valuable insights into the prevalence and characteristics of pediatric LCS, highlighting the significant quality of life and functioning impairment compared to control groups.
- By detecting neutrophilic granulocyte dysfunction in children with LCS, we shed light on a previously overlooked pathophysiological component of the condition.
- We demonstrate a significant correlation between clinical symptoms and superoxide production, further enhancing our understanding of the underlying mechanisms of pediatric LCS.

## Introduction

Coronavirus disease-2019 (COVID-19) received much attention since the WHO declared it a pandemic in March 2020.^1^ Over 760 million cases have been reported worldwide, and 60% of the pediatric population is assumed to have already been infected.^2,3^ Fortunately, children usually experience mild symptoms,^4^ presenting in ~40–60% of all cases with fever and cough.^5^ However, even mild cases may advance to complications like Multiorgan Inflammatory Syndrome in Children or long COVID syndrome (LCS).

The adult NICE guideline was among the first to define LCS. They described this condition as having new or ongoing symptoms persisting for ≥4 weeks after the onset of an acute infection.^6^ Though, the WHO later refined this definition to symptoms persisting for, or becoming apparent three months after the initial SARS-CoV-2 infection, lasting for at least two months without explanation. This new definition applied to adults^7^ and children^8^ as well. Regarding pediatric LCS with its specific features, a modified version after Delphi consensus^9^ has been developed for research. This stated that symptoms, of which at least one has to be physical, need to be experienced after a test-proven acute SARS-CoV-2 infection for a minimum of 12 weeks. These symptoms can impair the physical, mental, or social well-being of a child’s everyday life. Until this stricter definition, the lack of standardized case and control group interpretations led to a high level of heterogeneity^10^ for disease prevalence and reported symptom frequencies (1.6–70%).^11,12^ PCC prevalence in children was predicted to be 16.2% by a notable recent meta-analysis,^13^ placing the pooled prevalence estimates of various symptoms between 6.6% and 50.2%.^14^ Common symptoms include neuropsychological issues, loss of smell and taste, insomnia, gastrointestinal symptoms, and, outstandingly among children, upper airway symptoms (runny nose, cough).^14,15^ Mental symptoms (depression, anxiety) are shockingly common among children with LCS, but even healthy children were found to be susceptible to mental health issues during and after the pandemic.^16,17^ According to Stephenson’s CLoCk Study, the health-related quality of life was significantly lower among previously positive-tested children,^18^ but conflicting results exist.^19^

The research is extensive in the field of SARS-CoV-2 infection and its complications, and it forms an increasingly complex picture. The pathophysiology of the moderate or severe acute infection, during the hyper-inflammatory state, involves neutrophilic granulocytes with excessive neutrophil extracellular trap (NET) formation,^20^ leading to severe tissue damage, thrombophilia, multiorgan damage, and at times, death. Evidence from both adult and children studies showed that persisting SARS-CoV-2 viral particles might be able to cause mild chronic inflammation after an acute infection.^21–25^ Furthermore, according to a recent study, a significant association between this viral persistence and the presence of long COVID symptoms was observed in adults.^26^ Several immunological disruptions underpin this theory,^27–29^ and neutrophils appear to play a role in this disease as well.^30,31^ While elevated NET formation markers,^32^ raised the number of low-density neutrophils (subpopulation with enhanced ability to form NETs and produce proinflammatory cytokines),^33,34^ and neutrophil gene expression differences in the superoxide production^35^ were reported in adults, only elevated neutrophil count was reported in children with LCS.^36^ Despite the interest, to the best of our knowledge, neutrophil functions like superoxide production or phagocytosis in children with LCS have been greatly understudied.

Whilst examining children in our long COVID outpatient clinic, we felt the need to understand the pathology of LCS to find a distinguished feature helping us to diagnose LCS. Considering the observations of viral reactivation in COVID-19, the experienced role of neutrophils during the acute phase, and their disturbed activity’s pathogenic role in other diseases, we hypothesized an ongoing disruption in their functioning during COVID-19’s prolonged phase. Therefore, we planned to measure multiple neutrophil functions (Superoxide production, phagocytosis, NET formation, migration) and their influencing factors (IL-6, IFN-γ, IL-8). We intended to compare these outcomes between children with LCS and convalescent or uninfected participants to paint a complete picture of the functioning of these cells. Here, we 1) corroborate the previously described most frequent symptoms of LCS supplemented with the cardiovascular complaints; 2) show worse quality of life and functioning; and 3) present suppressed neutrophil responses in children suffering from LCS. However, whether the latter is a cause or a consequence of LCS remains an open question.

## Materials and methods

### Study design

Between 10 April 2021 and 10 April 2023, 140 children suffering from LCS, and 43 healthy children were enrolled in our case-control study at Semmelweis University, with the cooperation of the Physiology Institute and the Long COVID (LC) outpatient clinic of the Pediatric Department.

Data were collected before the appointment, through a standardized questionnaire (case report form—CRF),^37^ and during an in-person visit through clinical assessment and evaluation by a medical doctor. Children underwent standard-of-care examinations, and standardized laboratory testing was accomplished. At the first visit, additional blood samples were taken from 29 LCS patients and 17 healthy children, which were then analyzed in the Physiology Department.

Informed consent forms from the patients and parents were obtained. Venous blood was drawn following the Declaration of Helsinki, and according to the ethics permit reviewed by the Committee of Science and Research Ethics of the Medical Research Council (ETT TUKEB) and approved by the Department of Health Administration of the National Public Health Center of Hungary (Registration number: 34955-10/2021/EÜIG).

### Included population

We included three groups of children. The study group consisted of children with LCS (LCS group), recruited from the LC outpatient clinic of the Pediatric Department. To define the temporality of LCS symptoms, we utilized the NICE guideline as it was the currently valid guideline at the start of the study.^6^ As we in our previous publication, experienced, serious LCS could present within 3 months after the acute phase, and these children also need attention, even then. Therefore the Delphi consensus’s strict definition may be a rather limiting one in this aspect, and we decided to apply the NICE guideline henceforward. Acute COVID-19 was proven by polymerase chain reaction (PCR), lateral flow antigen, or antibody tests (anti-spike in non-vaccinated and anti-nucleocapsid in vaccinated children). Although a verified COVID-19 was not required by the NICE criteria, to increase the scientific value of our study, we only included children with a confirmed infection. We also excluded those, whose symptoms—after thorough examinations—were proven to be due to another condition (e.g., EBV infection, post-streptococcal disorder) or who sought us only for a health check after COVID-19 (*n* = 11).

The second and third groups constituted children who had not been infected with SARS-CoV-2 (COVID- uninfected control group, labeled as CG−, *n* = 8), and those who previously had COVID-19 but have not been suffering from abiding symptoms (COVID+ convalescent control group, labeled as CG+, *n* = 32). These children were recruited from the community, as healthy volunteers. The COVID status of these children was proven with the same methods as the LCS group, but we excluded 3 children because of borderline, thus inconclusive antibody test results. The minimum age of the CG was limited to 10 years, considering the need for informed consent from the self-volunteering healthy children.

From the above-detailed population, 29 children from the case group and 17 children from the CG+ control group, all above 10 years of age, were randomly selected for blood sampling, to test the neutrophil functions.

### Collected data

The applied guidelines and data collection were the same as those our research group published previously.^38,39^

The COVID-19 pandemic waves were assessed according to the Ourworldindata database^40^ and the information provided by the National Public Health Centre. The WHO’s classification^41^ was used to determine the severity of the acute SARS-CoV-2 infection.

A total of 50 questions about persisting symptoms since the SARS-CoV-2 infection (LCS and CG+) or in the last 4 months (CG−), were asked, with children (and their helping parents) giving positive (‘Yes, still present’; ‘Yes, intermittent’; ‘Yes, but not present anymore’) or negative (‘No’) answers. Symptoms were categorized into eight major organ system categories (general, cardiopulmonary, neurologic, mental, gastrointestinal, musculoskeletal, ear–nose–throat and ophthalmologic, and other—dermatological and reproductive) based on the categorization according to Garai et al. ^38^ The question “Erectile problems” were excluded, due to the absence of answers. Quality of life and functioning (QoL-F) in the last 7 days was measured by 12 elements on a 5-grade scale (max: 48 points), from “no difficulty” (0) to “extreme difficulty/cannot do” (4), where the higher scores represent a worse quality of life. These 12 functions were also compared between the situation before and after COVID-19 (LCS group, COVID+ control group), on a 3-point scale (better: −1 point, same: 0 point, worse: 1 point) to assess the QoL-F changes due to the infection itself. The higher sum of the points for the 12 functions was interpreted as worse functioning than before the infection (max: 12 points).

Pre-existing SARS-CoV-2 infection was proved by the detection of anti-spike antibodies with ELISA (Roche). Since the presence of viral nucleocapsid antibodies is not affected by vaccinations, for vaccinated children with unknown COVID status (*n* = 18), anti-nucleocapsid IgG was measured with ELISA with the help of the Department of Laboratory Medicine, Immune Laboratory subdepartment, Semmelweis University.

### Preparation of neutrophilic granulocytes from venous blood

Neutrophilic granulocytes (PMN) were separated by dextran sedimentation followed by Ficoll-Paque® gradient centrifugation, as previously described.^42^ After the first centrifugation, the blood plasma was also saved and stored at −80 °C for further analysis. Cells were finally resuspended in Hank’s Balanced Salt Solution (HBSS) in 10^6^ cells/mL concentration.

To test their viability, 1 × 10^5^ PMN cells were stained with 1% FITC Annexin V (BD Pharmingen^TM^) and with 1% propidium iodide (Life Technologies^TM^). The samples were incubated for 30 minutes at 37 °C with 400 RPM shaking in a thermo-shaker (BioSan Thermo-Shaker TS-100,), then centrifuged (500 × *g*, 10 minutes, 4 °C), and resuspended in 500 µl of ice-cold Phosphate-Buffered Saline (PBS).

The viability of the samples was measured with a flow cytometer (BECKMAN COULTER, CytoFLEX).

### Measurement of superoxide production

To measure superoxide production, 1.8 × 10^5^ primary human PMN cells were resuspended in HBSS containing lucigenin (Sigma-Aldrich), the latter was dissolved in dimethyl sulfoxide (Sigma-Aldrich) for a final concentration of 5 mg/mL. Cells were distributed into 96 well microplates (Greiner Bio-One, PS, F-Bottom, Chimney Well, White, Non-Binding) coated with 300 µl of 10% fetal bovine serum (FBS, Capricorn Scientific) for 1 hour at room temperature. Then, cells were stimulated with 5 mg/mL pooled human serum-opsonized zymosan^43^ or with 100 nM phorbol myristate acetate (PMA), and relative luminescence unit (RLU) was measured with a luminometer (BMG Labtech, CLARIOstar Reader, 90 cycles, 1 minute cycle time, 400 RPM double orbital shaking for 3 seconds before cycles, 37 °C), as described previously.^44^

### Determination of cytokine concentrations

For the measurement of neutrophil-derived IL-8, 6 × 10^5^ PMN cells were stimulated with 5 mg/mL pooled human serum-opsonized zymosan, or with 100 nM PMA for 4 hours at 37 °C with gentle shaking. Then, cells were centrifuged (500 × *g*, 10 minutes, 4 °C), and the IL-8 concentration of the supernatant was determined with a sandwich ELISA assay kit (R&D Systems DY208) using the protocol provided by the manufacturer. The absorbance was measured at room temperature using an ELISA plate reader (Labsystems iEMS Reader MF) (450 nm and 650 nm). The plasma concentrations of IL-6 and IFN-γ were also determined with sandwich ELISA assay kits (R&D Systems DY206-05, DY285B). For this, human plasma was separated after the first centrifugation (500 × *g*, 10 minutes) from venous blood.

### Investigation of neutrophil migration in vitro

The inserts of the Transwell® plate (Corning^®^ Costar^®^ Transwell Permeable Supports 6.5 mm Insert, 24 well plates, with 3-μm pore polycarbonate membrane insert, Sigma-Aldrich) were blocked with 10% FBS dissolved in HBSS (1 hour, 37 °C), and washed twice with HBSS. Then, 2 × 10^5^ PMN cells were added into the inserts, and migration was stimulated with the addition of 100 nM of the chemotactic agent N-formyl-methionyl-leucyl-phenylalanine (fMLP) into the bottom well. After incubation for 1 hour at 37 °C, the transmigrated cell count was determined with acid phosphatase assay.^45^

### Measurement of phagocytosis

The *Staphylococcus aureus* strain (USA 300), expressing green fluorescent protein (GFP) was a kind gift from Professor William Nauseef (University of Iowa). For the phagocytosis experiments, 10^9^ *S. aureus* was opsonized with pooled human serum for 20 min at 37 °C, with 600 RPM shaking. Then 6×10^5^ PMN cells were mixed with 6 × 10^7^ bacteria in a 1:100 ratio and incubated at 37 °C for 20 min. Samples (100 µl) were taken every 5 min and fixed with 4% (v/v) paraformaldehyde. Non-phagocytosed bacteria were quenched with the addition of 1% trypan blue solution (Sigma-Aldrich, 1:100 ratio), and the number of cells that engulfed at least one bacterium was measured with flow cytometry (Beckman Coulter, CytoFLEX). The median number of phagocytosed bacteria by PMN cells that phagocytosed, proportional to the green fluorescence intensity within the gate representing the phagocytosing cells, was also determined.

### Statistical analysis

Results were stored in REDCap^46,47^ electronic data capture tools hosted at Semmelweis University. Laboratory results were obtained from the institutional Medsol software (T-systems). Descriptive statistics were reported in mean, standard deviation, and relative frequencies. Normality and lognormality tests were performed to determine the distribution of the data. We considered a normal distribution if all the tests (Anderson-Darling test, D’Agostino & Pearson test, Shapiro-Wilk test, and Kolmogorov-Smirnov test) were significant. To examine group differences, in the case of normal distribution of our data, unpaired t-tests were performed, in other cases Mann–Whitney tests (simple or multiple) and ANOVA tests (one-way or two-way) were executed. Pearson’s chi-square or Fisher’s exact test was used to test the relationship between two categorical variables. If the assumption that the expected value of the cells should be 5 or greater in at least 80% of the cells and no cell should have an expected value less than 1 was not met, frequency data was reported without statistics. During this analysis, ‘cells’ refer to the cells containing frequency counts in contingency tables. To calculate correlation, we performed Spearman-Rank order correlation test. The level of significance was set a priori at 0.05. Statistical calculations were made by GraphPad Prism v9.5.0. and IBM SPSS Statistics 28.0.

## Results

### Demographic characteristics

We enrolled 140 children with LC symptoms in our study; then, after the exclusion of eleven cases, the number of participants in the LCS group was 129. The control group consisted of 40 children, of which 32 were convalescent COVID-19 (CG+), and 8 were uninfected (CG−). The mean ± SD age was 12.8 ± 3.4 in the LCS, 13.2 ± 2.5 in the CG+, and 13.8 ± 1.5 in the CG−. Age showed a non-significant difference between the groups (F(2,166) = 1.305, *p* = 0.290). The female sex ratio of the LCS and control groups, CG+ and CG− was 58.1%, 28.1%, and 62.5%, respectively. The prevalence of pre-existing conditions (chronic diseases, allergies, etc., detailed in Supplementary Table 2.) was 27.9% in the LCS group, 12.5% in the CG+ and 25% in the CG−. Children in the control groups, CG+ and CG−, were more often vaccinated against SARS-CoV-2 (53.1% and 87.5%) than those with LCS (27.9%), and in all cases, Pfizer-BioNTech COVID-19 Vaccine was used. Data are shown in Table 1. Further information about the vaccinations and their timely relation to COVID-19 and LCS are shown in Supplementary Table 3.

**Table 1:** Demographic characteristics and details of the acute SARS-CoV-2 infection of all examined children with and without long COVID symptoms.

			*n*/M ± SD	%	*p*
Number of children included	LCS		129		
	CG+		32		
	CG−		8		
Age (years)	LCS		12.8 ± 3.4		0.290
	CG+		13.2 ± 2.5		
	CG−		13.8 ± 1.5		
Age groups (years)	LCS	0–6	6	4.7	
		7–9	13	10.1	
		10–14	67	51.9	
		15–18	43	33.3	
	CG+	10–14	23	71.9	
		15–18	9	28.1	
	CG−	10–14	5	62.5	
		15–18	3	37.5	
Sex	LCS	Female	75	58.1	–^b^
		Male	54	41.9	
	CG+	Female	9	28.1	
		Male	23	71.9	
	CG−	Female	5	62.5	
		Male	3	37.5	
Pre-existing conditions	LCS		36	27.9	–^b^
	CG+		4	12.5	
	CG−		2	25.0	
Multiple SARS-CoV-2 infections	LCS		19	17.4	
	CG+		3	9.4	
Number of received SARS-CoV-2 vaccination^a^	LCS	0	92	71.3	–^b^
		1	3	2.3	
		2	32 (10)	24.8	
		3	2 (1)	1.6	
	CG+	0	15	46.9	
		2	8 (3)	25.0	
		3	9 (4)	28.1	
	CG−	0	1	12.5	
		2	6	75.0	
		3	1	12.5	
Diagnostic COVID tests	LCS	PCR	52	40.3	
		Ag	40	31.0	
		Spike Ab	37	28.7	
	CG+	PCR	6	18.8	
		Ag	10	31.2	
		Spike Ab - positive	8	25.0	
		Nucleocapsid Ab^c^ - positive	8	25.0	
	CG−	Spike Ab - negative	1	12.5	
		Nucleocapsid Ab^c^ - negative	7	87.5	
Acute COVID-19 severity	LCS	Without symptoms	6	4.7%	–^b^
		Mild	113	87.6%	
		Moderate	10	7.7%	
	CG+	Without Symptoms	6	25.0%	
		Mild	17	70.8%	
		Moderate	1	4.2%	
Time until the first visit (days)	LCS	From the COVID-19 (*n* = 124)	222.17 ± 133.72		0.869^d^
		From the onset of LC symptoms (*n* = 120)	196.72 ± 128.45		
	CG+	COVID-19 (*n* = 20)	247.65 ± 228.82		

^a^In parenthesis: the number of children who were vaccinated against SARS-CoV-2 before the acute COVID-19.

^b^The expected count of the cells containing frequency counts in contingency tables is less than 5.

^c^Only vaccinated children (*n* = 18).

^d^The difference between the two groups’ time from the COVID-19 until the first visit; *LCS* long COVID syndrome, *CG+* COVID-19 convalescent control group, *CG−* uninfected control group.

### Acute infection

**Fig. 1:**
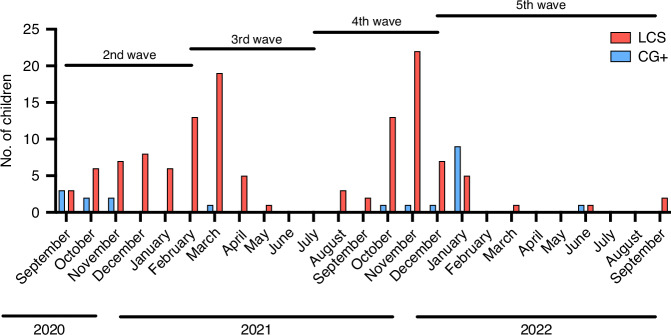
The monthly distribution of acute SARS-CoV-2 infection in the study population. The presence of SARS-CoV-2 in patients with long COVID syndrome (LCS) and in the control group was proved with PCR, rapid antigen test, or after the acute infection with antibody tests (anti-spike or anti-nucleocapsid ELISA). The *x* axis shows the timeline of the pandemic by month, and the y-axis shows the number of participants (LCS: 124, CG+: 20). The waves of COVID-19 in Hungary (elevation in the number of new cases/day) are indicated roughly. The third wave was dominated by the Alpha strain, the fourth by the Delta, and the fifth by the Omicron variant.

The COVID-19 waves and the time of acute illness of our study cohort are shown in Fig. 1. The majority with LCS had an acute infection in 2021 (*n* = 91, 73.4%). Whereas only 4 children in our convalescent control group were infected during this year (2021); the rest have fallen ill in 2020 (*n* = 6, 30%) or 2022 (*n* = 10, 50%). In the case of 5 children in the LCS and 12 children in the convalescent control group, the date of their acute illness is uncertain. Most of the children in both groups had a mild acute infection, and we observed a higher prevalence of the moderate disease course in the LCS group compared to the CG+. Asymptomatic infection occurred more often in the CG+ than in the LCS (Table 1).

The time frame between the acute infection and the in-person visit did not differ significantly between the LCS (mean time ± SD: 222.17 ± 133.72 days) and CG+ (mean time ± SD: 247.65 ±228.82 days) groups. Symptoms persisted continuously from the acute infection in 51% of children in the LCS group, and 38% experienced new symptoms in the first 3 months after their acute infection. After more than 3 months, 11% of children started to suffer from persisting symptoms (mean time ± SD of the onset of LCS symptoms from the end of the acute infection: 57.22 ± 49.29 days). The mean time ± SD from the appearance of the LC symptoms to the ambulatory care was 196.72 ± 128.45 days.

Table 1 contains data regarding the characteristics and the confirmation method of acute COVID-19.

### Long COVID symptoms and everyday complaints of healthy children

Inquiring after 50 symptoms typically occurring in LCS, every child of the LCS group answered positively at least once. Interestingly, 17 out of 32 children in the convalescent control group had a minimum of one complaint; the prevalence of a previously asymptomatic acute infection in this group was 46.9%. Even seven uninfected children (out of eight) without a history of COVID-19 had at least one complaint when asked. The number of experienced symptoms showed a significant difference between the groups. Children with LCS had significantly more out of the 49 symptoms than children in the CG+ and the CG−, with intermittently occurring symptoms being the most common in every group. Children with LCS had 5–10 times more symptoms on average, which were still present at the time of the visit than the CGs. Out of the “Yes” answers, already-resolved complaints were the least common for each group. Data are shown in Table 2.

**Table 2:** Comparison of the number of complaints (positive answers to a total of 50 questions) of children with long COVID syndrome to healthy children.

		min	max	M	SD	F	*p*	post hoc
All symptoms	LCS	1	34	11.80	7.35	32,962	<0.001	LCS > CG+= CG−
	CG+	0	17	3.47	4.55			
	CG−	0	15	5.13	5.28			
Yes, still present^a^	LCS	0	22	4.07	4.17	36,531	<0.001	LCS > CG+= CG−
	CG+	0	6	0.38	1.26			
	CG−	0	4	0.75	1.39			
Yes, intermittent^a^	LCS	0	27	6.34	4.98	13,849	<0.001	LCS > CG+
	CG+	0	11	2.53	3.24			CG+= CG−
	CG−	0	10	3.75	3.65			CG− = LCS
Yes, but not present anymore^a^	LCS	0	13	1.39	2.25	3711	0.044	LCS > CG+
	CG+	0	6	0.56	1.32			CG+= CG−
	CG−	0	5	0.63	1.77			CG− = LCS

^a^Answer to the question: “Have you noticed any of the mentioned symptoms after your acute COVID-19?”.

*LCS* long COVID syndrome, *CG+* COVID-19 convalescent control group; *CG−* uninfected control group.

**Fig. 2:**
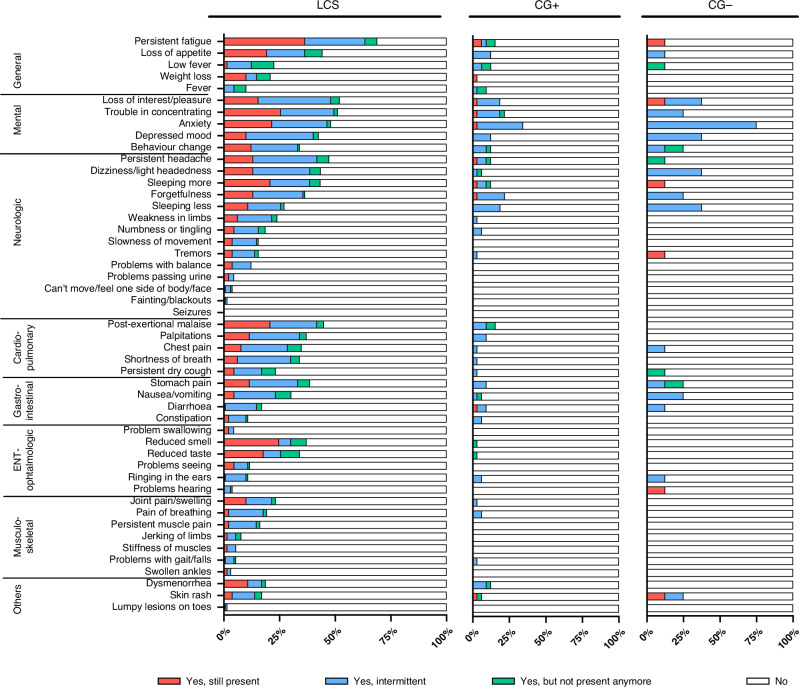
The distribution of symptoms between the long COVID (LCS) and control groups, detailed in the occurrence of symptoms. The detailed distribution of the frequency (the number of positive [“Yes, still present”, “Yes, intermittent”, “Yes, but not present anymore”] and negative “No” answers out of every given answer, depicted in percentage) of the 50 tested symptoms (49 shown, after erectile dysfunction was excluded due to lack of answers). Data was collected through an online questionnaire, based on the WHO’s standardized case report form^37^ confirmed and completed during an in-person meeting, from children with LCS (*n* = 129), healthy, COVID-19 convalescent (CG+: 32) and SARS-CoV-2 seronegative (CG−: 8) volunteers. The symptoms are grouped according to organ systems.

The LCS group’s most common symptoms were persistent fatigue (69%), loss of interest or pleasure (52%), and trouble concentrating (51.2%). Children in the convalescent control group most often reported anxiety (34.4%), forgetfulness (21.9%), and trouble concentrating (21.8%), and in the uninfected control group, anxiety (75%), sleeping less (37,5%) and loss of interest and pleasure (37,5%). We have found a statistically significant difference for 21 symptoms between the LCS and CG+ (Fig. 2). Every cardiovascular symptom occurred significantly more often in children with LCS than in the CG+ (Fig. 2, Suppl. Figs. 1 and 2).

### Quality of life and functioning

**Fig. 3:**
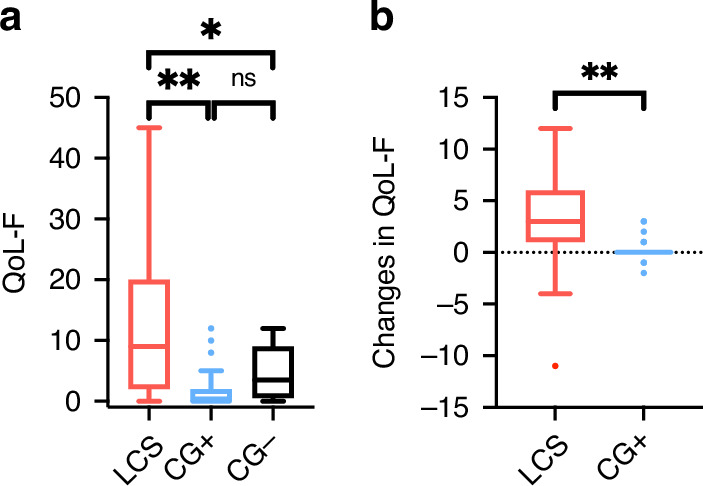
The total scores of the quality of life and functioning questionnaire and its changes compared to the situation before COVID-19. Through an online questionnaire based on the WHO’s standardized case report form ref. ^37^, the quality of life and functioning was scored on a 5-grade scale from “no difficulty” (0) to “extreme difficulty/cannot do” (4) (maximum sum of points 48) (**a**). Furthermore, the QoL-F items were compared to the situation before COVID-19 on a 3-point scale (better: -1 point, same: 0 point, worse: 1 point) to measure changes in QoL-F (max: 12 points) (**b**). Higher scores mean a worse quality of life in both cases. Mean ± SEM of 88 patients with long COVID syndrome (LCS) and 32 COVID+ and 8 COVID- controls were plotted. For statistical analysis, the Mann–Whitney *U* test was performed (*p < 0.05; **p < 0.01; ns: no significant difference).

The QoL-F questionnaire was fully completed by 89 children in the LCS and everyone in the control groups (*n* = 40). Patients in the LCS group reported a higher total score (worse functioning) (F(2,126) = 28.295, *p* < 0.001) than the convalescent (*p* < 0.001) and uninfected control group (*p* = 0.011) (Fig. 3a).

The changes in the QoL-F upon COVID-19 showed a significant difference between the LCS and CG+ (t(119) = 7.456, *p* < 0.001). The LCS exhibited markedly worse functioning following the onset of COVID-19 in contrast to the convalescent control group, wherein the majority of subjects displayed unaltered functioning. (Fig. 3b).

### Effect of Lzcs on superoxide production of neutrophilic granulocytes

**Fig. 4:**
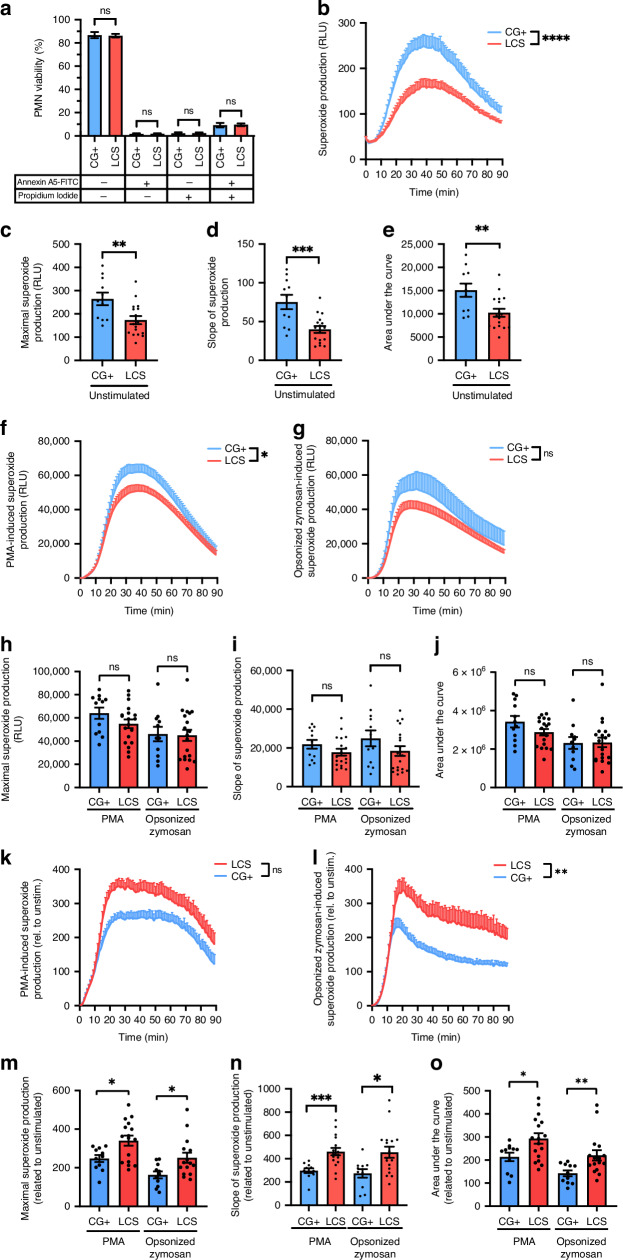
Viability and superoxide production of neutrophils isolated from patients with long COVID syndrome. The viability of neutrophilic granulocytes was tested with flow cytometry after Annexin A5-FITC (A5) and Propidium Iodide (PI) staining (**a**). The percentage (mean ± SEM of 27 [LCS], and 17 [control] independent experiments) of viable (double negative), early apoptotic (A5 + PI−), late apoptotic (double positive), or necrotic (A5-PI+) cells is shown. LCS: long COVID syndrome, n.s.: no significant difference. Mann–Whitney *U* test was performed. Basal superoxide production of PMN isolated from patients with long COVID syndrome (LCS) or healthy volunteers (control) (**b**). Unstimulated superoxide production was determined with lucigenin, and the relative luminescence unit (RLU) was measured in 90 minutes at 37 °C with a luminometer. The mean ± SEM of 17 (LCS) and 11 (control) independent experiments is plotted. Data were analyzed using a two-way repeated measures ANOVA. ****p < 0.0001 represents the significant interaction (time x disease). The maximal superoxide production of unstimulated PMN cells (**c**), the slope of the curves (**d**), and the area under the curve (**e**) were also determined. The mean ± SEM of 17 (LCS) and 11 (control) independent experiments is shown. Data were analyzed with an unpaired *t* test. **p < 0.01; ***p < 0.0001, compared to control. Superoxide production of neutrophilic granulocytes of children with long COVID syndrome (LCS) and healthy, COVID-19 convalescent (CG+) was measured after stimulation with PMA (**f**) or pooled human serum-opsonized Zymosan (**g**). Mean ± SEM of 19 (LCS) and 12 (control) independent experiments are plotted. Data were analyzed with two-way repeated measures ANOVA. *p < 0.05 represents the significant interaction (time x disease). n.s.: no significant interaction. The maximal superoxide production (**h**) the slope of the superoxide curves (**i**), and the area under the curves (**j**) are calculated. The mean ± SEM of 19 (LCS) and 12 (control) independent experiments (except **h** and **j**–control group Zymosan: 11) is plotted. Data were analyzed using a Mann–Whitney *U* test. n.s.: no significant difference. **k** PMA-, or pooled human serum-opsonized zymosan-induced superoxide production (**l**) normalized to the unstimulated samples. Mean + SEM of 17 (LCS) and 11 (control) independent experiments are plotted. Data were analyzed using two-way repeated measures ANOVA. **p < 0.01 represents the significant interaction (time x disease). n.s.: no significant interaction. Characteristics of the stimulated superoxide production—maximal superoxide production (**m**), the slope of the superoxide curves (**n**), and the area under the curve (**o**)–normalized to the unstimulated values, were calculated. Mean ± SEM of 17 (LCS) and 11 (control) individual experiments (except **m**—LCS group Zymosan: 15, PMA: 16, and **o**—LCS group Zymosan: 16) are shown. Statistical analysis was performed with the Mann-Whitney U test. *p < 0.05; **p < 0.01, compared to control.

For the analysis of neutrophilic granulocytes (polymorphonuclear cells, PMN), blood samples were taken from 29 children of the LCS, and 17 children of the convalescent control group. Theincluded children’s demographic and clinical data are shown in Suppl. Table 1. First, we investigated the viability of neutrophilic granulocytes isolated from LCS or control patients. Using Annexin A5 and Propidium Iodide staining, we did not find any significant difference between LCS and control cells: 86.21 ± 1.542% (LCS) and 86.77 ± 2.658% (control) of the cells were viable. Most of the non-viable PMNs (9.590 ± 1.190%, and 9.222 ± 1.937%, respectively) were Annexin A5—Propidium Iodide double positive necrotic cells (Fig. 4a). Although the resting, unstimulated superoxide production of neutrophils is relatively weak, its significant decrease was observed in the case of the LCS-derived cells compared to the neutrophils isolated from convalescent patients (Fig. 4b).

The individual RLU-time curves were evaluated based on three parameters: the maximum of the curve, i.e., the maximal superoxide production (Fig. 4c), the slope of the curve (Fig. 4d), and the area under the curve (Fig. 4e). All these three parameters were significantly decreased in the LCS group compared to the control, which points in the direction of reduced superoxide production of neutrophils isolated from LCS patients.

When LCS and control PMNs were stimulated with phorbol ester (PMA) or pooled human serum-opsonized zymosan, there was a marked increase in the production of superoxide. However, a noticeable disparity in the kinetics of superoxide production was only observed in the case of PMA stimulation Zymosan opsonization resulted in no significant difference compared to the control group (Fig. 4f, g). Analysis of the above-mentioned three parameters could not reveal any significant difference between the stimulated superoxide production of LCS and CG+ neutrophils (Fig. 4h–j). Normalization of the data to the basal superoxide production revealed that stimulating with PMA, the superoxide production of LCS PMNs was significantly higher. At the same time, upon zymosan stimulation, it was insignificant (*p* = 0.0557) compared to the control (Fig. 4k, l). Analyzing the characteristic parameters of the curves, we found ~1.3–1.5-fold change in favor of the LCS PMNs (Fig. 4m–o). These data suggest that upon stimulus, LCS neutrophils are capable of producing superoxide with a similar intensity as the healthy cells, however, their suppressed basal activity assumes a stronger response compared to the control cells.

### Phagocytosis of *Staphylococcus aureus* is impaired in LCS neutrophils

**Fig. 5:**
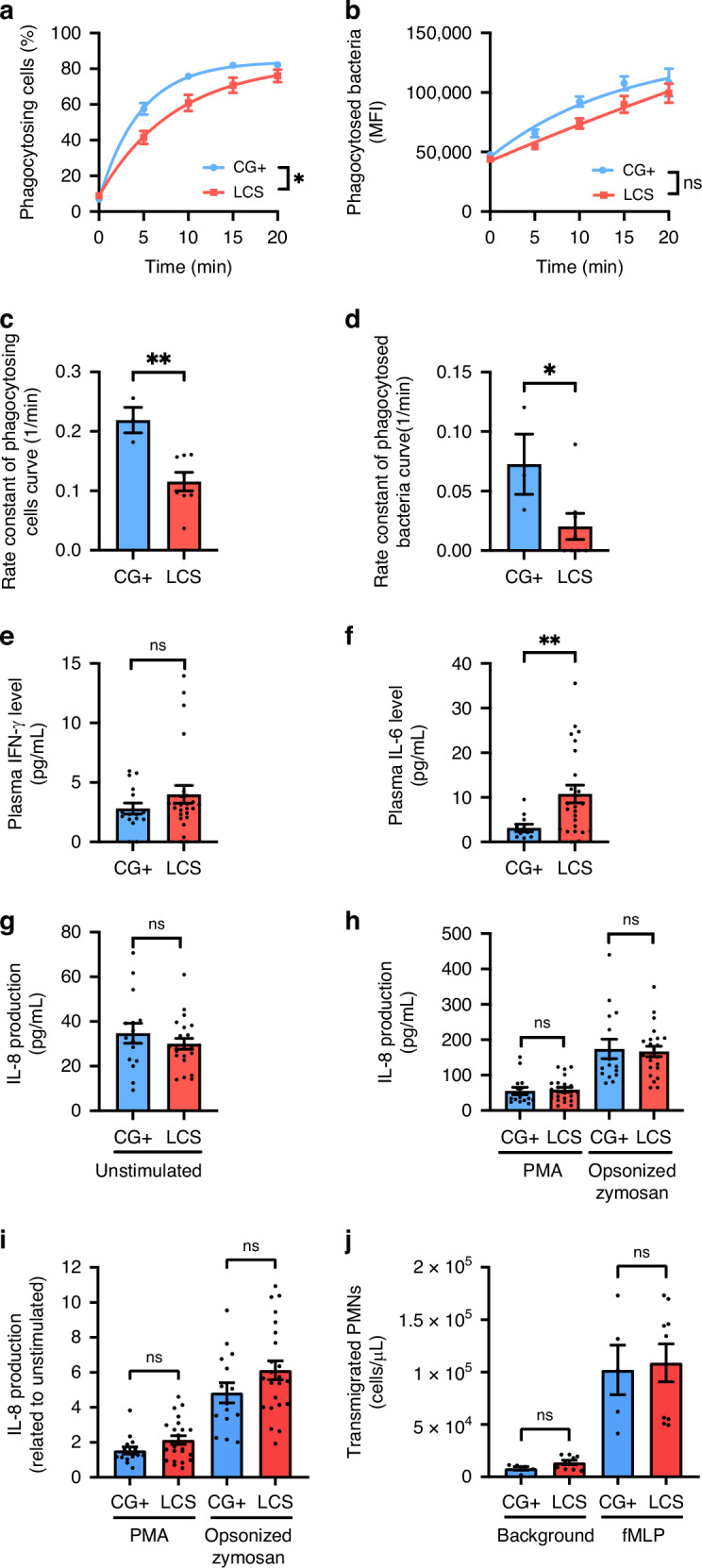
Phagocytosis, cytokine production, and migration of neutrophils isolated from patients with long COVID syndrome. PMN cells from LCS patients and healthy volunteers (control) were co-incubated with a green fluorescent protein (GFP) expressing *S. aureus* in a 1:100 ratio, and the kinetics of phagocytosis (**a**) and the number of phagocytosed bacteria (**b**) was determined with flow cytometry. The latter is proportional to the mean fluorescence intensity (MFI) measured in the FL1 (green) channel within the gate representing the phagocytosing cells. The mean ± SEM of 8 (LCS) and 4 (control) independent experiments is shown. Data were analyzed using two-way repeated measures ANOVA. *p < 0.05 represents the significant interaction (time x disease). n.s.: no significant interaction. The rate constants of the curves representing the bacteria-phagocytosing neutrophilic granulocytes (**c**) and the phagocytosed bacteria (**d**) were calculated. Mean ± SEM of 8 (LCS) and 3 (control) experiments are shown. A two-way repeated measures ANOVA was used for statistical analysis. *p < 0.05; **p < 0.01, compared to control. Plasma IFN-γ (**e**) (LCS: 25, control: 16) and IL-6 (**f**) (LCS: 24, control: 11) concentrations were determined with ELISA. The IL-8 production of LCS and control PMN cells was also determined in resting conditions (**g**) (LCS: 22, control: 15) or upon phorbol ester (PMA), as well as upon pooled human serum-opsonized Zymosan stimulation (**h**) (LCS: 22, control: 15). This data is also depicted after normalization to the unstimulated values (**i**) (LCS: 22, control: 15). Data were analyzed with the Mann–Whitney *U* test (**e**, **f**, **i**) or with unpaired *t* tests (**g**, **h**), mean ± S.E.M are plotted. **p < 0.01; ns: no significant difference, compared to control. Migration of the neutrophilic granulocytes of the LCS (*n* = 9) and control (*n* = 5) towards fMLP gradient in Transwell® assay. Transmigrated cell count was determined with an acid phosphatase assay (**j**). The two-way ANOVA and Šídák’s multiple comparisons tests were used for statistical analysis. Mean ± SEM are shown. n.s.: no significant difference compared to control.

Pooled human serum-opsonized, green fluorescence protein-expressing *S. aureus* bacteria were phagocytosed for 20 min, and kinetics of phagocytosis was measured. We found that the percentage of phagocytosing PMN cells was significantly decreased in the LCS group compared to the control group (Fig. 5a). Evaluating the number of phagocytosed bacteria of the cells that did phagocytose, which is proportional to the green fluorescence intensity within the gate of the phagocytosing PMN cells, we found no difference compared to the control (Fig. 5b). After performing a nonlinear regression analysis to model the saturation curves of phagocytosing cells and phagocytosed bacteria, the rate constants for both curves were determined to be significantly lower than those in the control group (Fig. 5c, d). These data suggest that besides superoxide production, phagocytosis of LCS neutrophils is also impaired.

### LCS affects IL-6, but not IFN-γ or IL-8 production

We measured the levels of IFN-γ and IL-6 in the blood plasma of patients with LCS and a control group. We also quantified the production of IL-8 by isolated PMNs. Our results showed no significant difference in IFN-γ levels in the plasma of LCS patients, but a significant elevation in plasma IL-6 concentration compared to the control group (Fig. 5e, f). Resting neutrophils obtained from both children with LCS and CG+ group produced similar levels of IL-8, with no significant difference even after stimulation with PMA or pooled human serum-opsonized zymosan (Fig. 5g, h). We also normalized the data to the basal IL-8 production, as a background, but this did not change our previous observation either and revealed no significant difference (Fig. 5i).

### LCS does not affect the migration of neutrophils in vitro

To determine the possible dysfunction in the migration of neutrophils toward chemoattractants, an in vitro Transwell^®^ assay was carried out. Neither the unstimulated nor the directed migration towards the fMLP gradient differed significantly compared to the control cells (Fig. 5j).

### Superoxide and IL-8 production correlate with the severity of LCS

**Fig. 6:**
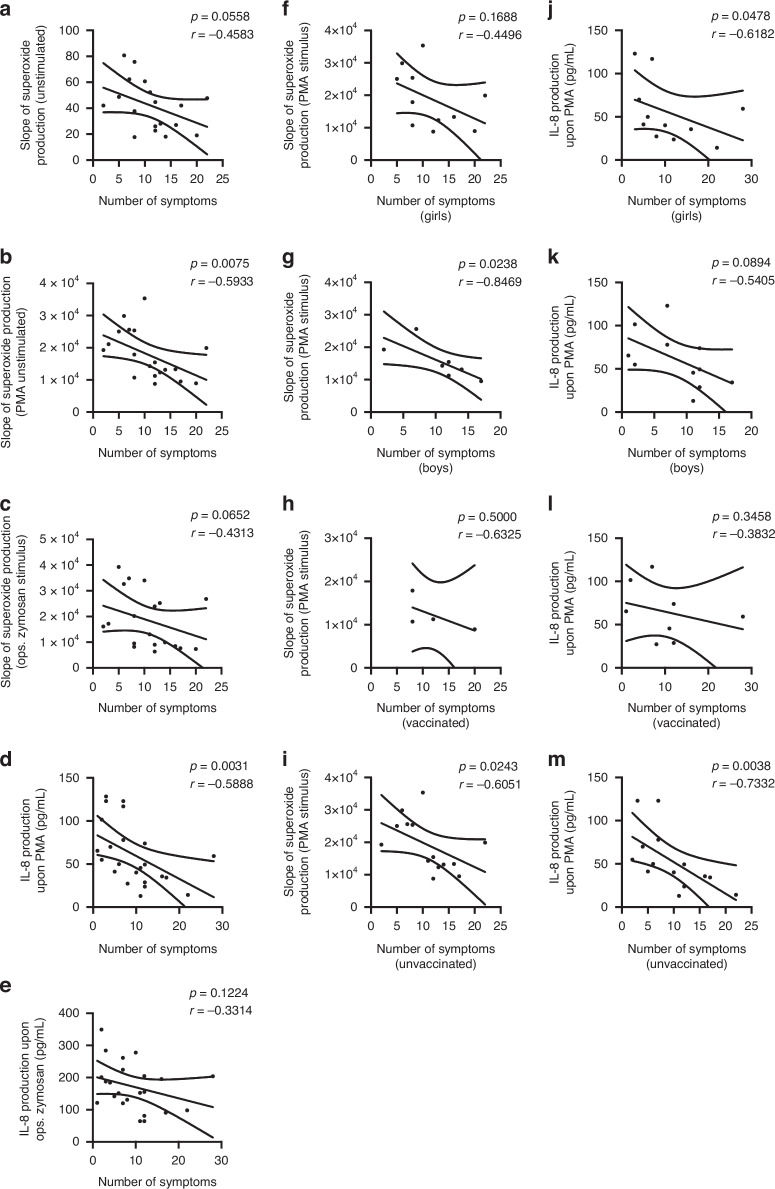
Correlation between the clinical data and laboratory results. Correlation analysis between the number of symptoms (out of 49) of children with long COVID syndrome (LCS) (*x* axis) and the slope of unstimulated superoxide production (*n* = 18) (**a**). Correlation between the number of symptoms and the PMA-induced superoxide production (*n* = 19) (**b**), as well as the pooled human serum-opsonized zymosan-induced superoxide production (*n* = 19) (**c**), were also calculated. IL-8 production of neutrophils isolated from LCS patients and stimulated with PMA (*n* = 23) (**d**) or stimulated with pooled human serum-opsonized zymosan (*n* = 23) (**e**) were also correlated with the number of symptoms. Correlation analysis between the slope of superoxide production upon PMA and the number of symptoms of girls (*n* = 11) (**f**) and boys (*n* = 7) (**g**) suffering from long COVID syndrome, or vaccinated (*n* = 4) (**h**), or unvaccinated children (*n* = 14) (**i**). Correlation between IL-8 production upon PMA and the number of symptoms of girls (*n* = 11) (**j**) and boys (*n* = 11) (**k**) suffering from long COVID syndrome, or vaccinated (n = 8) (**l**), or unvaccinated children (*n* = 14) (**m**). Data about the symptoms were collected through a standardized online questionnaire based on the WHO-made CRF.^37^ Unstimulated, PMA, or opsonized Zymosan-stimulated superoxide production was examined with a luminescent technique. Neutrophil-produced IL-8 levels after stimulation with PMA or opsonized Zymosan were measured with ELISA. The correlation was analyzed with the Spearman test.

Finally, we tested the potential correlation between the clinical and laboratory parameters to complete our work. Our tests revealed that the slope of the unstimulated (*n* = 18), as well as the PMA- and zymosan-stimulated (*n* = 19) superoxide production, correlated negatively with the number of symptoms (Unstim.: *r* = −0.4583; PMA: *r* = −0.5933; Zym.: *r* = −0.4313) (Fig. 6a–c). This result is significant only in the case of the neutrophils stimulated with PMA (*p* = 0.0074) (Fig. 6b), but not in the case of the unstimulated or Zymosan-stimulated state (Unstim.: *p* = 0.0558; Zym.: *p* = 0.0652) (Fig. 6a, c). Examining the subgroups of gender (female-male) and vaccination status (vaccinated-unvaccinated) (Fig. 6f–i), we found a significant negative correlation between the number of symptoms and the slope of superoxide production upon PMA stimulus of boys (*r* = −0.8469, *p* = 0.0238) (Fig. 6g) and the unvaccinated children (*r* = −0.6051, *p* = 0.0243) (Fig. 6i). Although we could not reveal any difference in the IL-8 production of the LCS’s and CG+’s resting or stimulated neutrophils (Fig. 5g–i), we found a negative correlation between the PMA-induced IL-8 levels and the maximum number of symptoms of children with LCS (*n* = 23, *r* = −0.5888, *p* = 0.0031). However, the stimulation with opsonized zymosan did not result in a significant difference (*r* = −0.3314 *p* = 0.1224) (Fig. 6d, e). Moreover, investigating the above-mentioned subgroups, we found that the number of symptoms and the PMA-stimulated IL-8 production of girls correlated significantly (*r* = −0.6182, *p* = 0.0478), but not of boys (*r* = −0.5405, *p* = 0.0894) and unvaccinated children (*r* = −0.7332, *p* = 0.0038). (Fig. 6j–m).

## Discussion

The present study compared 129 Hungarian pediatric patients suffering from LCS with 40 healthy children (convalescent and uninfected subgroups) as a control group. We analyzed the details of their SARS-CoV-2 infection as well as their long COVID symptoms and quality of life and functioning. Examining the WHO reported most frequent 50 symptoms (49 without erectile dysfunction), the most typical complaints were persistent fatigue and loss of interest.^10,38^ Besides these two, many other symptoms appeared significantly more frequent in the LCS group compared to the control group (e.g., persistent headache, reduced smell and taste, shortness of breath). The classification of the symptoms by organ systems revealed the strong involvement of cardiopulmonary complaints: all five, i.e., 100% of these symptoms differed significantly between the LCS and control groups. In contrast, only ~30% of the neurological symptoms and 60% of the ‘general’ (e.g., persistent fatigue, loss of appetite, fever, etc.) grouped symptoms showed a significant difference between LCS and convalescent control group. This implies the objectivity of the cardiovascular symptoms, though structural and functional alterations in LCS were not found by other researchers previously.^48,49^

Beyond the typical symptoms, we revealed an impaired quality of life and functioning in various fields of everyday life (e.g., learning, social interactions, experiencing new things) in the case of children with LCS. Even the QoL-F of convalescent and uninfected children was slightly affected. To examine whether the impairment is caused by the illness itself, not by personal difficulties or the psychological effect of the pandemic, we considered only those functions that have worsened since the acute infection and compared them to a healthily convalescent COVID-19 control group. We found that children with LCS have significantly worse quality of life and functioning since their acute COVID-19 than healthy children, so it is probable that the impairment is linked to their illness.

There are heterogeneous results in the literature considering the LCS’s symptomatology and the QoL-F of sick children. In contrast to many other studies, a notable limitation of our research is the relatively small sample size of participants, as it was challenging to recruit SARS-CoV-2 seronegative individuals, particularly children. Also, as we could only enroll children in our study who registered for a medical examination during ambulatory care, with a heterogeneously presenting condition, therefore the investigation of a homogenous group was impossible. Furthermore, creating matching control groups was demanding, hence the differences between the groups in sex, comorbidities, time to ambulatory care, and infection with varying variants. However, a significant strength of our study is the comprehensive evaluation of each child, including the assessment of their symptoms, quality of life, and functioning through questionnaires and in-person evaluations. Additionally, the inclusion of both a COVID-19 convalescent group and an uninfected control group in our study provides a detailed understanding of the symptomatology and quality of life among children with LCS.

The recruitment of neutrophilic granulocytes to the site of infection and their activation is an essential step of SARS-CoV-2 infection.^50^ According to their morphology and activation, recent studies classified the neutrophil population, which was previously believed to be homogenous, into subpopulations.^51–53^ The low-density granulocytes (LDGs) were reported as proinflammatory, NET-producing neutrophils colocalizing with peripheral blood mononuclear cells after Ficoll® gradient centrifugation, and found to be strongly involved in acute COVID-19 as well as in LCS.^34,50,51,54^ Here we provide evidence that activation and effector functions of normal-density neutrophils (NDNs), which sediment in Ficoll® gradient together with the erythrocyte fraction,^54^ are also altered in LCS: in resting conditions, spontaneous superoxide release of NDNs isolated from LCS children were decreased compared to control. Although PMA (a strong, direct activator of protein kinase C and used to achieve maximum superoxide production) could significantly increase the superoxide release of LCS NDNs, it neither reached nor exceeded the levels of healthy children. Similar results were obtained using pooled human serum-opsonized zymosan, which is a physiologically more appropriate stimulus. Additionally, phagocytosis of *S. aureus* by LCS NDNs was less effective compared to the control. Espín et al. suggested IFN-γ and IL-6, alongside others, as biomarkers of LCS.^55^ Our results on IL-6 are consistent with this finding, however, in the case of IFN-γ we found no difference in LCS patients compared to control. The observed disparity in results may be attributed to the relatively high -and therefore very dissimilar from ours- median age of 51.80 years among the population under study by Espín et al.

Although, recent studies evinced the involvement of NET in acute and post-acute COVID-19.^20,34,56–58^ Unfortunately, a limitation of our study, is that the relatively low neutrophil count obtained from children did not allow the investigation of NET.

Taken together, our data suggest an alteration in the NDN function, and remarkably, the dysfunction of the cells correlated with the illness severity of children with LCS: the more symptoms they had, the lower the stimulated superoxide and IL-8 production was. Many factors could influence the severity of LCS or the neutrophil responses, that aren’t fully understood to date. Also, as we mentioned above, our study group wasn’t perfectly homogenous, as LCS is a diverse disease, affecting different groups of people. Therefore, we created subgroups (girls and boys, vaccinated and unvaccinated children), and assessed our results. We haven’t found a difference in the number of symptoms between girls and boys or vaccinated and unvaccinated children (data not shown). Interestingly, the number of symptoms negatively correlated with the superoxide production upon PMA stimulus of boys and unvaccinated children, and the PMA-stimulated IL-8 level of girls and unvaccinated children. This demonstrates the impact of different characteristics of children on neutrophil responses. Furthermore, it raises questions about the influence of sex or the much-discussed effect of vaccination on LCS and its pathomechanism.

The dysfunction of the cells could be a phenotype predisposing to LCS, but we believe it to be rather a complication of the acute disease. Moreover, this may be a possible explanation for the virus reactivations (EBV, HHV6),^59^ the frequent Streptococcal tonsillitis,^60^ or upper airway infections of children with LCS observed in our praxis and worldwide with the more serious influenza or RSV seasons.^61^ Our limitations aside, our results offer compelling evidence for the possible pathological role of neutrophils in LCS and open new doors in the research of the disease in children.

## Supplementary information

Supplement Figs. 1 and 2

Supplemental Tables_Minor_Revision

## Data Availability

The original contributions presented in the study are included in the article/Supplementary Material. Further inquiries can be directed to the corresponding author.
