# Correction: Long COVID syndrome in children: neutrophilic granulocyte dysfunction and its correlation with disease severity

**DOI:** 10.1038/s41390-025-03888-3

**Published:** 2025-01-23

**Authors:** Fanni Kovács, Tamás Posvai, Eszter Zsáry, Ferenc Kolonics, Réka Garai, Vivien Herczeg, Domonkos Czárán, Johanna Takács, Attila József Szabó, Péter Krivácsy, Roland Csépányi-Kömi

**Affiliations:** 1 Pediatric Center, MTA Center of Excellence, Semmelweis University, Bókay Unit, Bókay János Street 53-54, 1083 Budapest, Hungary; 2 Department of Physiology, Semmelweis University, Budapest, Hungary; 3 Centre for Translational Medicine, Semmelweis University, Budapest, Hungary; 4 Department of Social Sciences, Faculty of Health Sciences, Semmelweis University, Budapest, Hungary

Correction to: *Pediatric Research* 10.1038/s41390-024-03731-1, article published online 27 November 2024

In this article the affiliation details for Author Réka Garai were incorrectly given as ‘Department of Social Sciences, Faculty of Health Sciences, Semmelweis University, Budapest, Hungary.’ but should have been ‘Centre for Translational Medicine, Semmelweis University, Budapest, Hungary’.

The affiliation details for Author Johanna Takács were incorrectly given as ‘Centre for Translational Medicine, Semmelweis University, Budapest, Hungary’ but should have been ‘Department of Social Sciences, Faculty of Health Sciences, Semmelweis University, Budapest, Hungary’.

The origianl article has been corrected.

