# Supplementary material for: Long COVID syndrome in children: neutrophilic granulocyte dysfunction and its correlation with disease severity
Source: Pediatr Res. 2024 Nov 27;98(1):301–13. doi: 10.1038/s41390-024-03731-1 (PMC12411219; doi:10.1038/s41390-024-03731-1)
Supplement: Supplementary file 1 — Supplement Figs. 1 and 2 [file 41390_2024_3731_MOESM1_ESM.pdf]

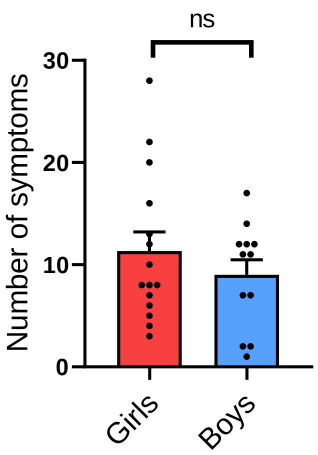

Supplement Figure 1: the number of symptoms between girls and boys involved in the neutrophil study. Mean+S.E.M. of n=15 (girls) and 12 (boys) were plotted ns: insignificant. Data were analyzed with an unpaired t-test.

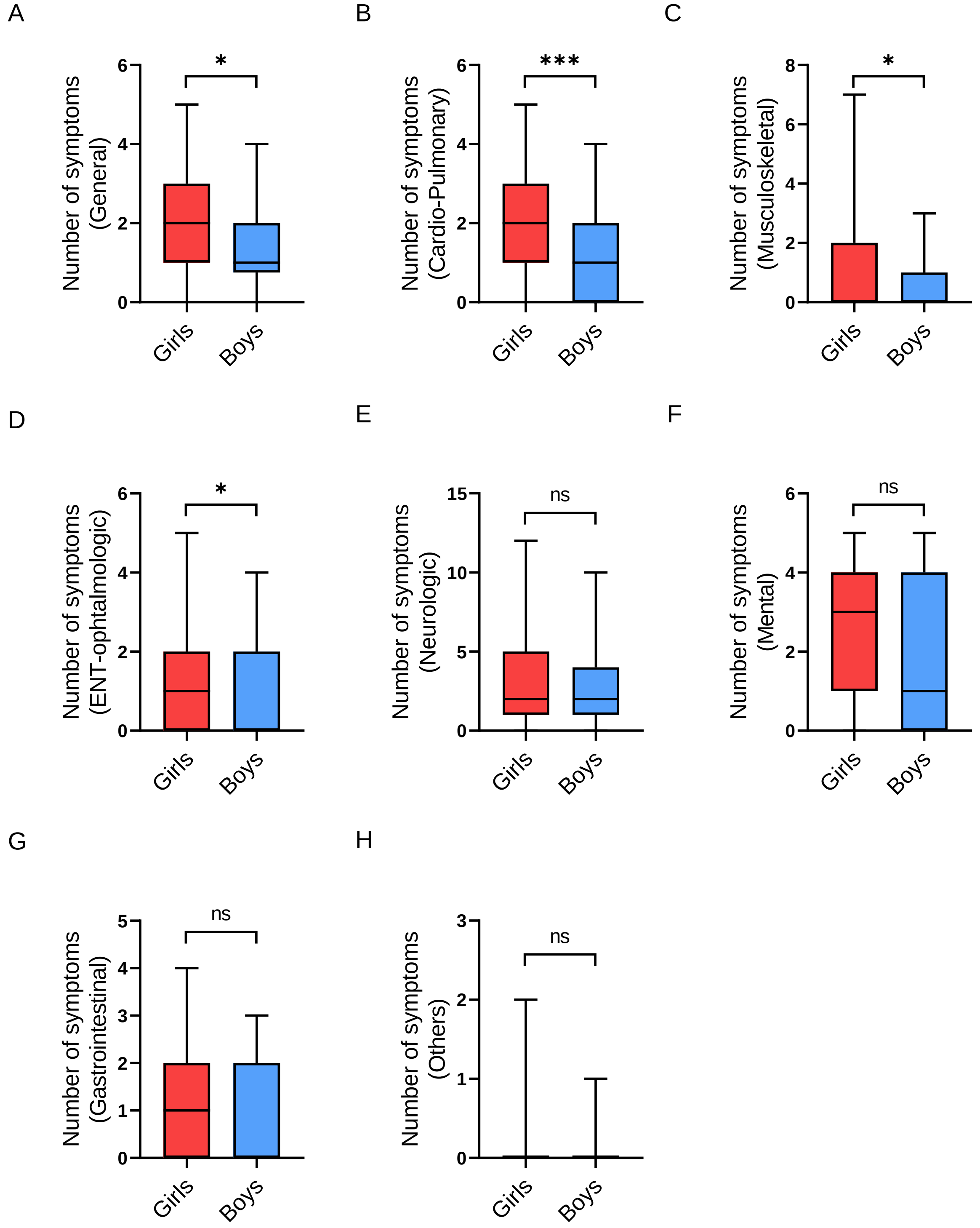

Supplement Figure 2: the number of symptoms grouped according to organ systems between girls and boys. Mean+S.E.M. of n=75 (girls) and 54 (boys) were plotted. *: $p<0.05$; **: $p<0.01$; ***: $p<0.001$; ns: insignificant. Data were analyzed with the Mann-Whitney U test.
